# A Comparative Study of Intra-articular Steroid Injection Alone Versus Steroid Injection Combined with Hydroplasty and Manipulation in the Treatment of Frozen Shoulder

**DOI:** 10.7759/cureus.105746

**Published:** 2026-03-24

**Authors:** Aman Thakur, Bhanu P Singh, Yogesh Singh Parihar

**Affiliations:** 1 Orthopaedics, Hindu Rao Hospital, New Delhi, IND; 2 Orthopaedics, Gajra Raja Medical College, Gwalior, IND; 3 Orthopaedics and Traumatology, Gajra Raja Medical College, Gwalior, IND

**Keywords:** frozen shoulder, hydroplasty, intrarticular steroids, methylprednisolone, shoulder pain and disability index (spadi)

## Abstract

Background

Frozen shoulder (adhesive capsulitis) is a common cause of shoulder pain and disability in middle-aged adults, characterized by progressive pain and restriction of both active and passive range of motion. Despite multiple available treatment modalities, the optimal minimally invasive intervention remains controversial. Intra-articular corticosteroid injection and hydroplasty with manipulation are widely practiced techniques, yet comparative evidence remains inconsistent.

Methods

This randomized comparative study was conducted at a tertiary care hospital over 18 months. Eighty patients aged 40-60 years with unilateral frozen shoulder refractory to conservative treatment were randomly allocated into two groups: Group A received intra-articular methylprednisolone injection, and Group B underwent intra-articular corticosteroid injection combined with capsular distension (hydroplasty) followed by controlled shoulder manipulation. Outcomes were assessed using the Shoulder Pain and Disability Index (SPADI) at baseline and at two weeks, six weeks, three months, 4.5 months, and six months. Statistical analysis was performed using IBM SPSS Statistics for Windows, Version 25 (Released 2017; IBM Corp., Armonk, New York, United States), with p < 0.05 considered statistically significant.

Results

Both groups demonstrated statistically significant improvement in SPADI scores across all follow-up intervals. Hydroplasty with manipulation showed significantly greater improvement at three months (p = 0.001) and 4.5 months (p = 0.026). At six months, outcomes were comparable between groups (p = 0.457). No major complications were observed.

Conclusion

Both intra-articular corticosteroid injection alone and corticosteroid injection combined with capsular distension and manipulation resulted in significant improvement in pain and functional disability in patients with frozen shoulder. Hydroplasty with manipulation demonstrated superior mid-term functional improvement; however, no statistically significant difference between the two treatment protocols was observed at six months of follow-up.

## Introduction

Frozen shoulder, also termed adhesive capsulitis, is a debilitating shoulder disorder characterized by progressive pain, stiffness, and global restriction of both active and passive glenohumeral movements. It affects approximately 2-5% of the general population and predominantly occurs in individuals between 40 and 70 years of age, with a higher prevalence in women and patients with metabolic disorders such as diabetes mellitus [1,2].

Although traditionally considered a self-limiting condition, long-term follow-up studies have demonstrated persistent pain, stiffness, and functional impairment in a significant proportion of patients, challenging the notion of spontaneous full recovery [3,4]. The disease typically progresses through three overlapping phases, freezing, frozen, and thawing, each associated with distinct clinical and pathological features [5].

Given the evolving pathophysiological characteristics across these phases, it may be hypothesized that treatment response could differ according to the disease stage. Intra-articular corticosteroid injection is expected to be more effective during the early “freezing” phase, where synovial inflammation and pain predominate, whereas hydroplasty, through mechanical capsular distension and manipulation, may offer greater benefits in the later “frozen” phase characterized by capsular fibrosis and contracture. However, comparative evidence evaluating a phase-specific therapeutic response remains limited, thereby justifying the present randomized comparison.

Histopathological studies have revealed fibroblastic proliferation, capsular thickening, and collagen deposition, particularly involving the rotator interval and coracohumeral ligament, suggesting that frozen shoulder is primarily a fibrotic rather than inflammatory disorder [6,7]. Elevated levels of profibrotic cytokines such as transforming growth factor-β and platelet-derived growth factor further support this hypothesis [8].

Management strategies for frozen shoulder include non-steroidal anti-inflammatory drugs, physiotherapy, intra-articular corticosteroid injections, hydrodilatation (hydroplasty), manipulation under anesthesia, and surgical capsular release [9]. Among these, minimally invasive interventions such as corticosteroid injections and hydroplasty are widely practiced due to their safety profile, cost-effectiveness, and feasibility in outpatient settings [10].

Intra-articular corticosteroids act by suppressing synovial inflammation and reducing pain, thereby facilitating rehabilitation, particularly in the early painful phase [11]. Hydroplasty, on the other hand, aims to mechanically distend the contracted joint capsule using saline with or without corticosteroids, theoretically addressing the underlying capsular fibrosis [12].

Despite multiple studies evaluating these modalities individually, comparative evidence remains inconsistent, especially regarding the timing, magnitude, and durability of functional improvement [13-15]. The objective of this randomized comparative study was to evaluate whether the addition of capsular distension (hydroplasty) and controlled manipulation to intra-articular corticosteroid injection provides superior clinical outcomes compared to corticosteroid injection alone in patients with frozen shoulder, as assessed by the Shoulder Pain and Disability Index (SPADI) [16].

## Materials and methods

Study site and design

This randomized comparative study was conducted in the Department of Orthopedics at Hindu Rao Hospital, Delhi, over a period of 18 months, from December 2020 to June 2022. Patients were recruited from the orthopedics outpatient department after obtaining written informed consent. The diagnosis of frozen shoulder was established on clinical grounds based on history and physical examination findings consistent with adhesive capsulitis. The study protocol was reviewed and approved by the Institutional Ethics Committee of Hindu Rao Hospital, Delhi. All procedures were conducted in accordance with the ethical standards of the institutional research committee and the principles of the Declaration of Helsinki. Written informed consent was obtained from all participants prior to enrolment.

Study population

A total of 80 patients, comprising both male patients and female patients aged between 40 and 60 years, were enrolled in the study. All included patients had unilateral frozen shoulder and had failed to respond to a minimum of three weeks of conservative management, which included non-steroidal anti-inflammatory drugs, muscle relaxants, heat therapy, and supervised physiotherapy exercises. Only patients who provided informed consent were included. Enrolment commenced in December 2020, and eligible participants were randomly allocated into two equal groups of 40 patients each.

Sample size justification

Sample size estimation was performed using the SPADI as the primary outcome measure. A difference of 10 points in the SPADI score between the two groups was considered clinically significant. Assuming a standard deviation of 16.9, a two-sided alpha error of 0.05, and a power of 80%, the minimum calculated sample size was 32 patients per group. Accounting for an anticipated 10% loss to follow-up, a final sample size of 35 patients per group was required. To further strengthen statistical reliability, 40 patients were included in each group.

Randomization and group allocation

Eligible participants were randomly allocated into two groups in a 1:1 ratio using a computer-generated randomization sequence prepared prior to study initiation. Allocation was performed using sequentially numbered, opaque, sealed envelopes to ensure concealment of group assignment until the time of intervention. The envelopes were opened only after patient enrolment and baseline assessment had been completed. This process minimized selection bias and ensured unbiased group allocation.

Inclusion and exclusion criteria

Patients were included if they had unilateral frozen shoulder defined as greater than 50% limitation of passive range of motion compared to the contralateral side in one or more planes of movement (abduction, forward flexion, or external rotation at 0° abduction) with a hard end feel, symptom duration of at least three months, age between 40 and 60 years, and controlled diabetes mellitus (HbA1c 6-7%).

Patients were excluded if they had uncontrolled diabetes mellitus, prior manipulation of the affected shoulder under anesthesia, inflammatory or degenerative rheumatologic disorders involving the shoulder, fracture or dislocation of the affected shoulder, rotator cuff injury, previous shoulder surgery, Hill-Sachs lesion, severe osteoporosis, malignancy involving the shoulder region, neurological deficits affecting shoulder function, known allergy to bupivacaine, pregnancy or lactation, or a history of corticosteroid injection in the affected shoulder within the preceding four weeks.

Pre-procedure evaluation

All patients were counseled in their native language regarding the nature of the disease, details of the procedure, expected outcomes, and possible complications. Baseline demographic data, including age, sex, occupation, and side involved, were recorded. A detailed clinical history, including duration of symptoms and previous treatments, was obtained. Routine pre-procedural investigations included complete blood count, random blood sugar, bleeding time, clotting time, and prothrombin time-international normalized ratio. An anteroposterior radiograph of the affected shoulder was obtained to exclude other pathologies.

Study tools and outcome measures

Data collection was performed using a structured case reporting form. Clinical outcomes were assessed using the SPADI, a validated instrument measuring pain and functional disability in shoulder disorders. The validated standard version of the SPADI, as originally described in the literature, was used for outcome assessment.

Intervention protocol

All procedures were performed under strict aseptic precautions with the patient in a sitting position at the edge of the bed, with the arm adducted by the side and the elbow flexed and forearm supinated. The skin was prepared with povidone-iodine solution.

In Group A, patients received an intra-articular injection of 2 mL of methylprednisolone acetate (80 mg) mixed with 3 mL of 0.5% bupivacaine using a 25-gauge, 1.5-inch needle via the posterior approach. The injection site was identified approximately 2 cm inferior and medial to the posterolateral corner of the acromion. The needle was advanced anteromedially toward the coracoid process to a depth of 3-4 cm. Aspiration was performed before injection to avoid intravascular administration. After injection, patients were advised supervised physiotherapy.

In Group B, hydroplasty with manipulation was performed using the same posterior approach. The injected solution consisted of 2 mL of methylprednisolone (80 mg), 3 mL of 0.5% bupivacaine, and 15 mL of normal saline. Approximately 20 minutes after injection, gentle manipulation of the affected shoulder was performed with the patient in a sitting position. This included gradual forward elevation with scapular stabilization, passive external rotation at 0° and 90° of abduction, internal rotation at 90° of abduction, and cross-body adduction. Care was taken to apply controlled force to avoid complications. Post-procedure, patients underwent supervised physiotherapy. 

Both groups received intra-articular methylprednisolone; the distinguishing component of Group B was capsular distension (hydroplasty) followed by controlled manipulation.

Following the procedure, both groups were instructed to follow an identical standardized physiotherapy protocol consisting of pendulum exercises, cross-body stretching, finger-walk exercises, and internal and external rotation exercises. Patients were advised to perform exercises twice daily at home and were reviewed at follow-up visits for reinforcement and correction of technique. However, adherence to physiotherapy was not objectively quantified.

Follow-up and outcome evaluation

Patients in both groups were followed up at two weeks, six weeks, and subsequently at six-week intervals up to six months. At each visit, SPADI scores were recorded, and patients were assessed for adherence to physiotherapy exercises, which were reinforced and corrected as required.

Data management and statistical analysis

Categorical variables were expressed as frequencies and percentages, while continuous variables were presented as mean ± standard deviation or median with interquartile range, as appropriate. Intergroup comparisons of continuous variables were performed using the independent t-test, and intragroup comparisons across follow-up intervals were analyzed using the paired t-test. Categorical variables were analyzed using the Chi-square test or Fisher’s exact test when expected cell values were less than five. Data entry was performed using Microsoft Excel, and statistical analysis was conducted using IBM SPSS Statistics for Windows, Version 25 (Released 2017; IBM Corp., Armonk, New York, United States). A p-value of less than 0.05 was considered statistically significant.

## Results

A total of 80 patients with clinically diagnosed unilateral frozen shoulder were enrolled and randomized equally into two treatment groups: Group A (intra-articular methylprednisolone injection) and Group B (hydroplasty with manipulation). All patients completed the six-month follow-up period, and no cases were lost to follow-up.

Baseline characteristics

Baseline demographic and clinical characteristics were comparable between the two groups. There was no statistically significant difference in age distribution, sex, side involved, or occupational profile (p > 0.05), confirming adequate randomization and minimizing confounding bias. The mean baseline SPADI score was 70.08 ± 13.33 in Group A and 74.15 ± 11.44 in Group B, with no statistically significant difference (p = 0.146), indicating comparable baseline pain and disability severity.

Primary outcome: SPADI

Intergroup Comparison Over Time

Both treatment modalities resulted in a progressive and statistically significant reduction in SPADI scores over successive follow-up intervals.

Early follow-up (two and six weeks): At two weeks, mean SPADI scores reduced to 55.92 ± 12.31 in Group A and 58.28 ± 10.46 in Group B. The difference was not statistically significant (p = 0.360). At six weeks, SPADI further reduced to 44.30 ± 8.58 in Group A and 44.12 ± 7.46 in Group B (p = 0.923). These findings indicate equivalent short-term efficacy of both interventions in reducing pain and disability.

Mid-term follow-up (3 and 4.5 months): At three months, Group B demonstrated significantly lower SPADI scores (29.25 ± 7.28) compared to Group A (34.52 ± 6.83), with a statistically significant difference (p = 0.001). This superiority of hydroplasty with manipulation persisted at 4.5 months, where SPADI scores were 20.85 ± 5.30 in Group B versus 23.62 ± 5.60 in Group A (p = 0.026).

Late follow-up (six months): At six months, SPADI scores converged, measuring 15.12 ± 4.03 in Group A and 14.42 ± 4.33 in Group B, with no statistically significant difference (p = 0.457). 

Interpretation

Hydroplasty with manipulation provided statistically superior mid-term functional improvement, whereas long-term outcomes were comparable between both treatment modalities (Table 1).

**Table 1:** Intergroup Comparison of SPADI Scores at Different Follow-Up Intervals SPADI: Shoulder Pain and Disability Index

Time Point	Steroid (Mean ± SD)	Hydroplasty with Manipulation (Mean ± SD)	p-Value
Baseline	70.08 ± 13.33	74.15 ± 11.44	0.146
Two weeks	55.92 ± 12.31	58.28 ± 10.46	0.360
Six weeks	44.30 ± 8.58	44.12 ± 7.46	0.923
Three months	34.52 ± 6.83	29.25 ± 7.28	0.001
4.5 months	23.62 ± 5.60	20.85 ± 5.30	0.026
Six months	15.12 ± 4.03	14.42 ± 4.33	0.457

Intra-group analysis of SPADI

Group A: Intra-Articular Methylprednisolone Injection

In Group A, patients treated with intra-articular methylprednisolone injection demonstrated a progressive and statistically significant reduction in SPADI scores at all follow-up intervals when compared with baseline. The mean pre-intervention SPADI score was 70.08 ± 13.30, indicating substantial pain and functional disability at presentation. At the first follow-up at two weeks, the mean SPADI score significantly decreased to 55.92 ± 12.31 (paired t-test, p < 0.0001), reflecting early pain relief and functional improvement. This improvement continued at six weeks, with the mean SPADI score reducing further to 44.30 ± 8.58 (p < 0.0001). At three months, a sustained reduction was observed, with a mean SPADI score of 34.52 ± 6.83, which remained statistically significant (p < 0.0001). At 4.5 months, the mean SPADI score decreased to 23.62 ± 5.60, and by six months, it further reduced to 15.12 ± 4.03, indicating marked clinical improvement with near-resolution of symptoms. All reductions from baseline across follow-up visits were statistically significant (p < 0.0001), confirming the effectiveness of intra-articular corticosteroid injection in reducing pain and disability over time (Table 2).

**Table 2:** Intragroup Comparison of SPADI Scores in Group A (Methylprednisolone) Data expressed as mean ± SD; paired t-test used for intra-group comparison; p < 0.05 considered statistically significant. SPADI: Shoulder Pain and Disability Index

Follow-up Interval	Mean ± SD	Median (IQR)	Range	95% CI of Mean Difference	p-Value
Pre-operative	70.08 ± 13.30	72.5 (62–79.25)	41–94	–	–
Two weeks	55.92 ± 12.31	59 (47.5–64)	29–82	13.221 to 15.079	<0.0001
Six weeks	44.30 ± 8.58	45 (40.25–51)	24–57	23.839 to 27.711	<0.0001
Three months	34.52 ± 6.83	35 (31–41)	17–45	32.85 to 38.25	<0.0001
4.5 months	23.62 ± 5.60	23 (21–28.25)	11–32	43.397 to 49.503	<0.0001
Six months	15.12 ± 4.03	14 (13–18.25)	6–22	51.688 to 58.212	<0.0001

Group B: Hydroplasty with Manipulation

Group B patients who underwent hydroplasty with manipulation also showed a consistent, statistically significant decline in SPADI scores at each follow-up compared to baseline. The mean baseline SPADI score was 74.15 ± 11.44, indicating severe pain and disability prior to intervention. At two weeks, the mean SPADI score significantly decreased to 58.28 ± 10.46 (paired t-test, p < 0.0001). Further improvement was noted at six weeks, with the mean SPADI score reducing to 44.12 ± 7.46 (p < 0.0001). At 3 months, a pronounced reduction was observed, with the mean SPADI score declining to 29.25 ± 7.28, demonstrating sustained functional recovery (p < 0.0001). This improvement persisted at 4.5 months, with a mean SPADI score of 20.85 ± 5.30, and at six months, the score further decreased to 14.42 ± 4.33. All reductions from baseline were highly statistically significant (p < 0.0001), indicating that hydroplasty with manipulation resulted in substantial and durable improvement in pain and shoulder function (Table 3).

**Table 3:** Intragroup Comparison of SPADI Scores in Group B (Hydroplasty with Manipulation) Data expressed as mean ± SD; paired t-test used for intra-group comparison; p < 0.05 considered statistically significant. SPADI: Shoulder Pain and Disability Index

Follow-up Interval	Mean ± SD	Median (IQR)	Range	95% CI of Mean Difference	p-Value
Pre-operative	74.15 ± 11.44	75 (67.75–82.25)	45–94	–	–
Two weeks	58.28 ± 10.46	61 (52–67.25)	32–78	14.808 to 16.942	<0.0001
Six weeks	44.12 ± 7.46	43 (41.75–49.5)	27–62	27.982 to 32.068	<0.0001
Three months	29.25 ± 7.28	28.5 (24.5–34.25)	16–44	41.566 to 48.234	<0.0001
4.5 months	20.85 ± 5.30	21 (17–22)	11–32	49.95 to 56.65	<0.0001
Six months	14.42 ± 4.33	14 (11.75–15.25)	8–27	56.427 to 63.023	<0.0001

Within-group analysis using paired t-tests demonstrated a highly significant reduction in SPADI scores from baseline at all follow-up intervals in both groups (p < 0.0001). Group A showed a 78.4% reduction in the mean SPADI score at six months. Group B demonstrated an 80.5% reduction in the mean SPADI score at six months. These findings confirm that both interventions are clinically effective in the management of frozen shoulder.

Safety outcomes

No major complications such as infection, fracture, neurovascular injury, or post-injection flare requiring intervention were observed in either group. Minor transient pain at the injection site was noted in both groups and resolved spontaneously.

## Discussion

The present randomized comparative study demonstrates that both intra-articular corticosteroid injection alone and corticosteroid injection combined with capsular distension and controlled manipulation significantly improve pain and functional disability in patients with frozen shoulder. The combined intervention was associated with superior mid-term functional recovery, whereas long-term outcomes at six months were comparable between groups.

Demographic and clinical correlation

The age distribution in this study (mean 48.68 years) aligns with prior epidemiological data indicating peak incidence in the fifth decade of life [1-3]. The lack of significant sex predilection is consistent with several large observational series, although some studies report a slight female predominance [4]. The absence of baseline differences between groups strengthens the internal validity of the study and supports the reliability of comparative outcome analysis.

Interpretation of early outcomes

The comparable early improvement observed at two and six weeks reflects the anti-inflammatory and analgesic effect of corticosteroids, which rapidly reduce synovitis and pain, facilitating physiotherapy [9,11]. Hydroplasty with manipulation also demonstrated early benefit, likely due to capsular stretching and immediate joint volume expansion. This finding is consistent with existing evidence suggesting that both modalities are effective during the painful “freezing” phase of frozen shoulder [5,10].

Mid-term superiority of hydroplasty with manipulation

The mid-term superiority observed in the intervention group likely reflects the combined mechanical and pharmacologic effects of capsular distension and controlled manipulation in addition to corticosteroid therapy. Given the composite nature of the intervention, it is not possible to attribute the observed benefit exclusively to capsular distension alone [6,7]

Long-term outcome convergence

By six months, SPADI scores were comparable between groups, supporting the concept that frozen shoulder is ultimately a self-limiting condition, with gradual resolution occurring irrespective of intervention [3,4]. Therefore, within the six-month follow-up period of the present study, no statistically significant difference was observed between the two treatment techniques in terms of final functional outcome. This convergence has been previously reported and suggests that the primary benefit of intervention lies in accelerating recovery and reducing morbidity, rather than altering the ultimate outcome [2,8].

Clinical implications

Steroid injections may be preferred in early, painful stages where rapid pain relief is required. Hydroplasty with manipulation may be advantageous in patients with established stiffness or those not responding adequately to steroid injection. Both interventions are safe, cost-effective, and suitable for outpatient settings.

Study limitations

The present study has several limitations that should be considered when interpreting the findings. First, the study was conducted at a single tertiary care center, which may limit the external generalizability of the results. Patient demographics, disease characteristics, and treatment responses may vary across institutions and healthcare settings. Multicenter studies involving more diverse populations would help validate the reproducibility of these findings.

Second, injections were performed using anatomical landmark techniques without ultrasound or fluoroscopic guidance. Although landmark-guided injections reflect common clinical practice, the absence of imaging confirmation may introduce variability in intra-articular placement of the injectate, potentially influencing treatment response.

Third, the intervention in the hydroplasty group consisted of a composite procedure including capsular distension and controlled manipulation in addition to corticosteroid injection. Because manipulation was not performed in the steroid-only group, the relative contribution of capsular distension and manipulation to the observed mid-term improvement cannot be independently determined. This represents a potential confounding factor in the intergroup comparison.

Fourth, clinical outcomes were assessed using the SPADI, a validated patient-reported outcome measure. However, objective assessment of shoulder range of motion using standardized goniometric measurements was not included. The absence of objective biomechanical assessment limits the ability to correlate symptomatic improvement with structural recovery.

Fifth, although a standardized physiotherapy program was recommended to both groups, adherence and progression were not objectively quantified. Variability in compliance with rehabilitation exercises may have influenced functional outcomes.

Finally, the follow-up duration was limited to six months. While this period allowed evaluation of short- and mid-term outcomes, longer follow-up would be necessary to assess the durability of treatment effects and potential late recurrence of symptoms.

Despite these limitations, the randomized comparative design, complete follow-up of all participants, and use of a validated clinical outcome measure strengthen the internal validity of the study. Future studies incorporating multicenter recruitment, imaging-guided injections, objective range-of-motion assessment, and longer follow-up durations may further refine treatment strategies for adhesive capsulitis.

## Conclusions

Both intra-articular corticosteroid injection alone and corticosteroid injection combined with capsular distension and manipulation resulted in significant improvement in pain and functional disability in patients with frozen shoulder. The combined intervention demonstrated superior mid-term improvement in SPADI scores at three and 4.5 months. However, at six months, outcomes between the two treatment protocols were statistically comparable, indicating no significant long-term difference between the interventions. These findings suggest that while hydroplasty with manipulation may accelerate functional recovery in the intermediate period, both treatment approaches ultimately achieve similar clinical outcomes by six months. Future multicenter studies incorporating imaging-guided injection techniques, objective range-of-motion measurements, and longer follow-up durations are warranted to further refine treatment strategies.
